# Appetitive Olfactory Learning and Long-Term Associative Memory in *Caenorhabditis elegans*

**DOI:** 10.3389/fnbeh.2017.00080

**Published:** 2017-05-01

**Authors:** Saori Nishijima, Ichiro N. Maruyama

**Affiliations:** Information Processing Biology Unit, Okinawa Institute of Science and Technology Graduate UniversityOkinawa, Japan

**Keywords:** chemotaxis, extinction learning, massed and spaced trainings, olfactory conditioning, short-term and long-term memories

## Abstract

Because of the relative simplicity of its nervous system, *Caenorhabditis elegans* is a useful model organism to study learning and memory at cellular and molecular levels. For appetitive conditioning in *C. elegans*, food has exclusively been used as an unconditioned stimulus (US). It may be difficult to analyze neuronal circuits for associative memory since food is a multimodal combination of olfactory, gustatory, and mechanical stimuli. Here, we report classical appetitive conditioning and associative memory in *C. elegans*, using 1-nonanol as a conditioned stimulus (CS), and potassium chloride (KCl) as a US. Before conditioning, *C. elegans* innately avoided 1-nonanol, an aversive olfactory stimulus, and was attracted by KCl, an appetitive gustatory stimulus, on assay agar plates. Both massed training without an intertrial interval (ITI) and spaced training with a 10-min ITI induced significant levels of memory of association regarding the two chemicals. Memory induced by massed training decayed within 6 h, while that induced by spaced training was retained for more than 6 h. Animals treated with inhibitors of transcription or translation formed the memory induced by spaced training less efficiently than untreated animals, whereas the memory induced by massed training was not significantly affected by such treatments. By definition, therefore, memories induced by massed training and spaced training are classified as short-term memory (STM) and long-term memory (LTM), respectively. When animals conditioned by spaced training were exposed to 1-nonanol alone, their learning index was lower than that of untreated animals, suggesting that extinction learning occurs in *C. elegans*. In support of these results, *C. elegans* mutants defective in *nmr-1*, encoding an NMDA receptor subunit, formed both STM and LTM less efficiently than wild-type animals, while mutations in *crh-1*, encoding a ubiquitous transcription factor CREB required for memory consolidation, affected LTM, but not STM. The paradigm established in the present study should allow us to elucidate neuronal circuit plasticity for appetitive learning and memory in *C. elegans*.

## Introduction

Learning and memory are essential for animals to survive and reproduce in ever-changing environments. Appetitive conditioning is a form of associative learning and is the process by which a new predictive relationship between a cue (or action) and a reward is learned. During such conditioning, a stimulus acquires new motivational significance through association with the reward. Understanding appetitive conditioning is important for elucidating mechanisms of both learning and motivational processes (for reviews, see Martin-Soelch et al., 2007; Fanselow and Wassum, 2016). Appetitive conditioning has been demonstrated in many organisms, including *Aplysia*, dogs, *Drosophila*, honeybees, humans, rats, and snails (e.g., Pavlov, 1927; Alexander et al., 1984; Bouton and Peck, 1989; Schwaerzel et al., 2003; Giurfa et al., 2009; Austin and Duka, 2010; McDannald et al., 2011; Michel et al., 2011; Nargeot and Simmers, 2011; Burke et al., 2012; Eisenhardt, 2014; Andreatta and Pauli, 2015).

The major advantage of invertebrates for the study of learning and memory is the relative simplicity of their nervous systems. *Caenorhabditis elegans* is also an excellent model for studies of appetitive conditioning. Hedgecock and Russell (1975) found that *C. elegans* grown at 16, 20, or 25°C with food migrates to its growth temperature and then moves isothermally, suggesting that the animal associates its cultivation temperature with the presence of food, and remembers the association for several hours. In contrast, starved experience induced aversive responses to cultivation temperatures (Hedgecock and Russell, 1975; Mohri et al., 2005), although other recent studies have failed to find evidence of long-term association between temperature and presence or absence of bacterial food (Chi et al., 2007; Kobayashi et al., 2016). *C. elegans* is also able to form an association between the odorant benzaldehyde and the food content in its environment (Nuttley et al., 2002). Torayama et al. (2007) also reported that chemotaxis of *C. elegans* to butanone is enhanced by pre-exposure of the animal to the chemical in the presence of food. Based on this discovery, positive olfactory associative assays were designed to study learning and memory in *C. elegans* (Kauffman et al., 2010; Stein and Murphy, 2014).

The nervous system of *C. elegans* hermaphrodites has completely been reconstructed from serial electron micrographs of thin sections. Its 302 neurons form ∼7,000 chemical synapses and ∼600 gap junctions (White et al., 1986). The body is transparent throughout life, from fertilized egg to adult, so neural activities of living animals can be observed using genetically modified fluorescent proteins sensitive to voltage or Ca^2+^ concentration. *C. elegans* detects numerous volatile and water-soluble chemicals as attractants and repellents, mainly through its amphid sensilla (Ward, 1973; Dusenbery, 1974; Bargmann and Horvitz, 1991), and modulates its behavior based on experience (Hobert, 2003; Sasakura and Mori, 2013). Amphids are the largest chemosensory organs in *C. elegans*, and each one consists of 12 sensory neurons with ciliated dendrites, as well as a sheath and a socket glial cell (Ward et al., 1975; Ware et al., 1975). Amphid neurons serve various functions, including chemotaxis, thermotaxis, mechanosensation, osmotaxis, and dauer pheromone sensation (Bargmann and Mori, 1997; Driscoll and Kaplan, 1997; Riddle and Albert, 1997; de Bono and Maricq, 2005; Bargmann, 2006). Chemotaxis of *C. elegans* to cations, anions, cyclic nucleotides, and amino acids was first described by Ward (1973), and since then this list has been extended to include many olfactory stimuli (Bargmann et al., 1993). For example, 1-nonanol is a weak repellent for *C. elegans* (Bargmann et al., 1993), while potassium chloride (KCl) is a strong attractant (Ward, 1973). Both K^+^ and Cl^-^ ions are mainly sensed by a single sensory neuron, ASER (Ortiz et al., 2009).

As described above, appetitive conditioning has exclusively been demonstrated using food as an unconditioned stimulus (US) in *C. elegans*. Partly because behavior of *C. elegans* is dramatically affected by the presence or absence of food (Gray et al., 2005), the distinction between associative learning and non-associative learning, which includes sensitization, habituation, and adaptation, is not clear (Bargmann, 2006). Rather than pairing chemical cues with food, which is a gustatory, olfactory and mechanical stimulus all in one, it would be preferable to use two defined chemical cues for conditioning *C. elegans* in order to analyze neuronal networks responsible for memory traces. In the present study, we developed a paradigm to study appetitive olfactory conditioning and associative memory in *C. elegans*. Under this paradigm, we conditioned animals with 1-nonanol as a conditioned stimulus (CS), and KCl as a US. Spaced training with an intertrial interval (ITI) induced long-term memory (LTM), while massed training without an ITI induced short-term memory (STM) which was disrupted by cold shock. The formation of LTM, but not STM was dependent on mRNA and protein synthesis, and required activity of genes shared by other model organisms, including *Aplysia, Drosophila*, and mice.

## Materials and Methods

### Strains Used

The wild-type Bristol N2 and mutant strains, *crh-1(tz2)* and *nmr-1(ak4)*, used in this study were obtained from the *Caenorhabditis* Genetics Center (University of Minnesota, Minneapolis, MN, USA). *stau-1(tm2266)* was from the National Bioresource Project for the Nematode (Tokyo Women’s Medical University School of Medicine, Japan). *glr-1(ky176)* was a generous gift from Andres Maricq (University of Utah, Salt Lake City, UT, USA). Animals were grown to adulthood on nematode growth medium (NGM) (50 mM NaCl, 2% agar, 2.5% peptone, 1.0 mM cholesterol, 1.0 mM CaCl_2_, 1.0 mM MgSO_4_, 25 mM potassium phosphate, pH 6.0) seeded with *Escherichia coli* OP50 under unstarved conditions at 20°C using standard methods (Brenner, 1974).

### Chemotaxis Assay

**FIGURE 1:**
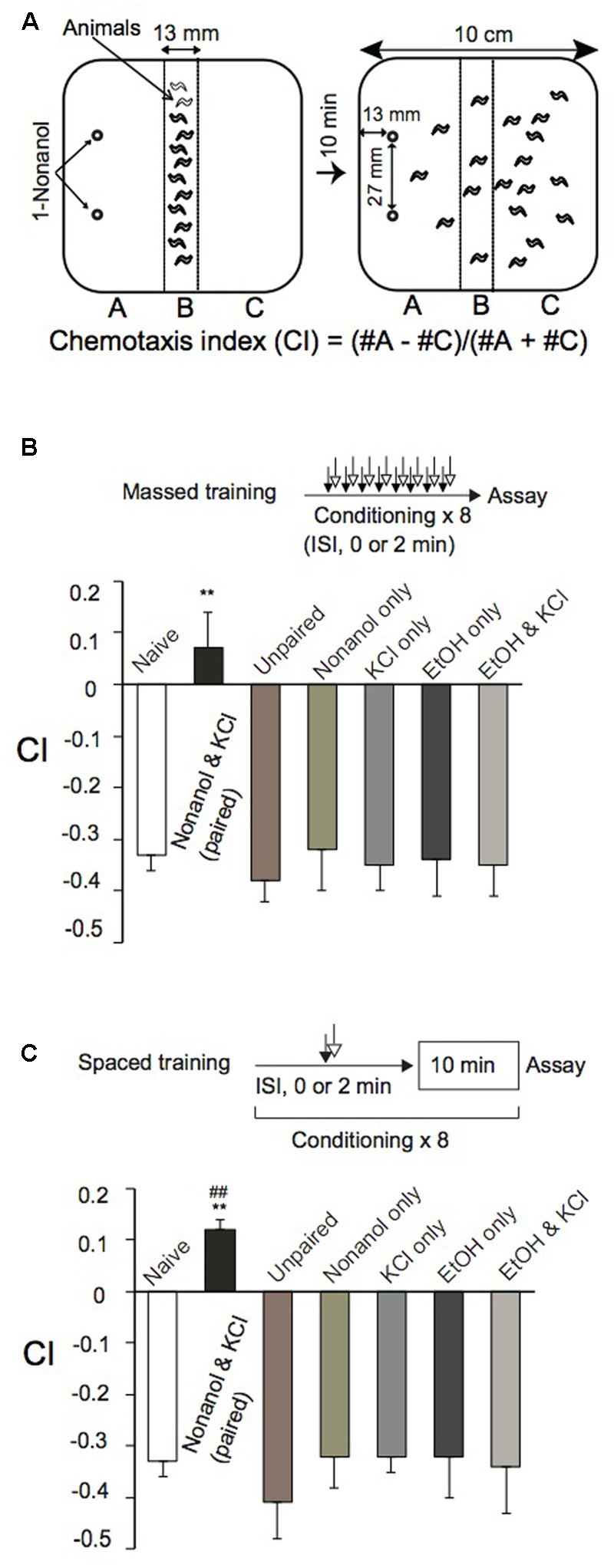
**Classical conditioning of *Caenorhabditis elegans*. (A)** Chemotaxis assay. After conditioning, an olfactory chemotaxis assay was performed on a square agar plate. Approximately 150 animals were placed along the central line of the plate. Then, 3 μl of 0.01% 1-nonanol solution diluted with EtOH was spotted at two places along the edge of the plate as shown. After 10-min incubation at RT, animals were killed with chloroform vapor. CI was calculated using the equation shown, after counting the number of animals in sections “A” and “C.” **(B,C)** Massed training and spaced training. Animals were conditioned eight times by massed training **(B)** or spaced training **(C)** with chemicals indicated. Paired training with 1-nonanol and KCl, or EtOH and KCl, was carried out consecutively without an intervening delay (ISI, 0 s), while unpaired training with 1-nonanol and KCl was conducted consecutively with ISI (120 s). Chemotaxis assays were performed immediately after conditioning. Note that CIs of reference animals trained by massed training or spaced training with 1-nonanol and KCl (unpaired; ISI, 120 s), 1-nonanol only, KCl only, EtOH only, or EtOH and KCl were statistically indistinguishable from that of naïve animals. Asterisks (^∗∗^) indicate statistically significant differences (^∗^*p* < 0.01) determined by one-way ANOVA, followed by the Tukey-Kramer test for further comparison with CIs of unpaired, control animals. CIs of animals conditioned by spaced training **(C)**, but not those conditioned by massed training **(B)**, with 1-nonanol and KCl (paired) were statistically (^##^*p* < 0.01) different from the “0” base line when analyzed by using one sample *t-*test. Data are displayed as mean ± SEM (*n* = 9 assays).

For olfactory chemotaxis assays, we used a 10-cm square plate containing 14 ml of 1.5% agar, 1.0 mM CaCl_2_, 1.0 mM MgSO_4_, and 5 mM potassium phosphate (pH 6.0). Animals were collected by washing them off NGM plates with 0.25% aqueous gelatin solution, and were gently washed three times with ∼1.0 ml 0.25% aqueous gelatin by decantation. Approximately 150 animals were placed along the central line of the agar plate, and gelatin solution was then removed as much as possible with a Kimwipe wick. Then, 3 μl each of 0.01% 1-nonanol diluted with 100% ethanol (EtOH), unless otherwise stated, were spotted at two places along the edge of the plate (**Figure 1A**). After covering the plate with a lid, animals were allowed to move freely for 10 min at room temperature (RT), and were then killed by placing a drop of chloroform on the lid. Animals in areas “A” and “C” (**Figure 1A**) were counted to calculate a chemotaxis index (CI) using the equation shown in **Figure 1A**.

### Resource Localization Assay

Potassium chloride localization assays were performed using 10-cm Petri dishes divided into four quadrants, as described previously (Wicks et al., 2000) with modifications (Supplementary Figure S1A). Two adjacent quadrants were filled with agar (2% agar, 10 mM HEPES (pH 7.2), 1.0 mM CaCl_2_, and 1.0 mM MgSO_4_) supplemented with or without KCl, ranging from 5 to 200 mM (Supplementary Figure S1B). In quadrants without KCl, sorbitol solution was added to adjust their osmolality (10–350 mOSM) to that of quadrants with KCl. Osmolality of solutions was measured using an OSMOMAT osmometer (model 030-D; Gonotec, Berlin, Germany). Agar plates in adjacent quadrants were connected by placing a thin layer of 2% molten agar on top of the plastic separators. Animals were collected from an NGM plate by washing them off the plate in 0.25% aqueous gelatin solution, and after washing the animals three times with ∼1.0 ml aqueous gelatin solution (0.25%), approximately 100 animals in ∼100 μl of 0.25% aqueous gelatin solution were placed at the intersection of the four quadrants. A Kimwipe wick was used to remove as much gelatin solution as possible. After 30 min incubation at RT, animals were killed by placing a drop of chloroform on the lid. Animals in each quadrant were counted to calculate performance index (PI), dividing the number of animals on quadrants containing KCl by the total number of animals on the entire plate (Supplementary Figure S1A).

### General Conditioning

**FIGURE 2:**
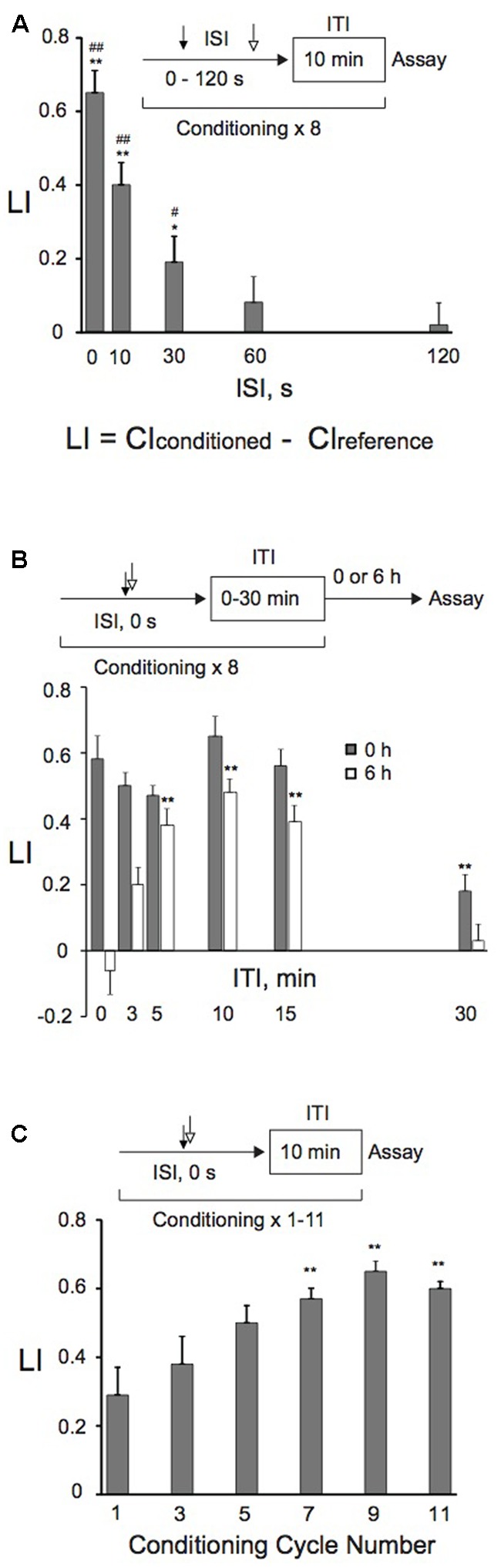
**Effects of ISI and ITI lengths, or conditioning cycle numbers on memory retention. (A)** Effects of ISI lengths on memory formation. Animals were conditioned with 0.01% 1-nonanol and 160 mM KCl consecutively without an intervening delay (0 s), or 10, 30, 60, or 120 s later with 160 mM KCl after the end of 1-nonanol stimulation by eight-cycle spaced training with a 10-min ITI. Chemotaxis assays were carried out immediately after conditioning. Asterisks (^∗^*p* < 0.05, ^∗∗^*p* < 0.01) indicate statistically significant differences determined by one-way ANOVA, followed by the Tukey-Kramer test for further comparison with LI values of animals conditioned with ISI (120 s). LIs of animals conditioned with various ISIs were statistically (^#^*p* < 0.05, ^##^*p* < 0.01) different from the “0” base line when analyzed by using one sample *t-*test. An equation for calculation of learning index (LI) is also shown, and CI_reference_ is CI of animals treated first with 100% EtOH and then with 160 mM KCl by massed or spaced training unless otherwise stated. Bars are means ± SEM (*n* = 6–9 assays). **(B)** ITI effects on memory formation and retention. Animals were conditioned with 0.01% 1-nonanol and 160 mM KCl consecutively without an intervening delay (ISI, 0 s), using eight-cycle spaced training with indicated ITI lengths. Chemotaxis assays were performed immediately (gray bars) or 6 h (open bars) after conditioning. Asterisks (^∗∗^) indicate statistically significant differences (*p* < 0.01) determined by one-way ANOVA, followed by the Tukey-Kramer test for further comparison with LIs of animals conditioned without an ITI. LI values were calculated from the equation shown in **(A)** using CI_reference_ values of animals treated with 100% EtOH and 160 mM KCl. Bars are means ± SEM (*n* = 6–9 assays). **(C)** Effects of the number of conditioning cycles on memory formation. Animals were conditioned with 0.01% 1-nonanol and 160 mM KCl consecutively without an intervening delay (ISI, 0 s), using a 10-min ITI and repeating the conditioning 1–11 times. Immediately after conditioning, chemotaxis assays were performed. Asterisks (^∗∗^) indicate statistically significant differences (*p* < 0.01) determined by one-way ANOVA, followed by the Tukey-Kramer test for further comparison with LIs of single-conditioned animals. LI values were calculated from the equation shown in **(A)** using CI_reference_ values of animals treated with 100% EtOH and 160 mM KCl. Bars are means ± SEM (*n* = 9 assays).

1-Nonanol solution (0.01%, 30 ml, diluted with 100% EtOH) was placed in a 500-ml beaker, and a small plastic dish, 6 cm in diameter, containing 10 ml of 160 mM KCl was placed on a plastic stand, 1.5 cm high, in the beaker. Well-fed animals 4 days after hatching were collected from an NGM plate by washing the plate three times with ∼1.0 ml aqueous gelatin solution (0.25%). Then animals were transferred to a transparent plastic pipe (polymethylmethacrylate; 1.5 cm long, 3 cm external diameter, 2-mm wall thickness), the bottom of which was closed with a nylon mesh sheet (pore size; 30 μm). Animals in the plastic container were lowered into the beaker slowly (∼10 s) to be stimulated with 1-nonanol vapor as a CS, and then briefly (<1.0 s) with KCl as a US. Animals were washed once by gently immersing the container in an excess (100 ml) of doubly deionized water (ddH_2_O; Millipore Elix 10 UV, followed by Millipore Milli-Q Synthesis A10, Merck Millipore), and an excess amount of water was removed by placing the container on a paper towel. The container was then placed on a chemotaxis assay plate for 10 min, unless otherwise stated, for spaced training as described below. For massed training, this resting process was omitted. This procedure was repeated eight times unless otherwise stated, and fresh ddH_2_O was used each time for washing, so as to prevent contamination. Then, animals were collected in a 1.5 ml tube by suspending them in ∼1.0 ml of 0.25% aqueous gelatin, and were used immediately for chemotaxis assay, or were incubated on an NGM plate with an OP50 lawn at RT until post-training chemotaxis assay. A learning index (LI) was calculated by subtracting CI of reference animals mock-conditioned with 100% EtOH and 160 mM KCl from that of conditioned animals (**Figure 2A**). LI values in **Figures 5, 6** were calculated by subtracting of CI of unpaired (ISI, 120 s) animals stimulated with 1-nonanol and KCl from that of paired (ISI, 0 s) animals.

### Conditioning with Various Interstimulus Intervals

An interval between stimulations, an interstimulus interval (ISI), with 0.01% 1-nonanol diluted with EtOH and 160 mM KCl was varied, ranging from 0 s to 2 min. In the present study, ISI is a period of time between the end of CS stimulation and the on-set of US stimulation. Animals in a plastic container were lowered into a beaker saturated with vapor from 0.01% 1-nonanol diluted with EtOH for ∼10 s, and were gently washed with ddH_2_O (100 ml), followed by resting on a chemotaxis assay plate until a designated period of time. Then animals were immersed in 160 mM KCl solution briefly (<1.0 s), and were gently washed by immersing the animals in ddH_2_O (500 ml). After removing excess water by placing the container on a paper towel, animals were rested on a chemotaxis assay plate for 10 min at RT until the next cycle of conditioning. This conditioning was repeated eight times. In case of “ISI, 0 s,” animals were lowered into a beaker saturated with vapor from 0.01% 1-nonanol and 100% EtOH for approximately 10 s, and were then dipped into 160 mM KCl without washing with ddH_2_O.

### Conditioning with Various Intertrial Intervals

Animals were consecutively conditioned with 0.01% 1-nonanol, which was diluted with EtOH, and 160 mM KCl as described above in Section “General Conditioning,” and were gently washed with ddH_2_O. For massed training, animals were subjected to the next cycle of conditioning without rest. For spaced training, animals in a plastic container rested on a chemotaxis assay plate for 10 min, unless otherwise stated, before the next conditioning. Conditioning was repeated eight times unless otherwise stated. After completion of conditioning, animals were washed with ∼1.0-ml 0.25% aqueous gelatin solution, and were either subjected to the chemotaxis assay or incubated on an NGM plate with an OP50 lawn at 20°C until post-training chemotaxis assay. All other aspects of conditioning, testing, and scoring were as described above in Section “General Conditioning”.

### Cold Shock

Immediately after conditioning, animals in a container were gently immersed in ice-cold ddH_2_O for 5 s. Excess water was removed by placing the container on a paper towel, and animals were either subjected to chemotaxis assay or incubated on an NGM plate with an OP50 lawn at 20°C until post-training chemotaxis assay. All other aspects of conditioning, testing, and scoring were as described above in Section “General Conditioning”.

### Extinction Learning

After “general spaced training” (ISI, 0 s; ITI, 10 min; eight cycles), animals were incubated on an NGM plate with an OP50 lawn for 3 h at 20°C. Then, animals were collected by washing them off the plate with 0.25% aqueous gelatin solution (∼1.0 ml), and were conditioned with only CS by slowly (∼10 s) lowering the container into a 500-ml beaker saturated with vapor of 0.01% 1-nonanol diluted with EtOH. Animals were gently washed by immersing the container in ddH_2_O (100 ml), and then the container was placed on a paper towel to remove excess water. This conditioning was repeated eight times without an ITI. Chemotaxis assays were performed immediately after conditioning, and all other aspects of conditioning, testing, and scoring were as described above in Section “General Conditioning”.

### Inhibitor Treatment

Nematode growth medium agar plates containing inhibitors were prepared by mixing molten NGM agar with 0.3 μg/ml cycloheximide (Sigma, Saint Louis, MO, USA), 0.3 μg/ml anisomycin (A.G. Scientific, San Diego, CA, USA), or 0.1 μg/ml actinomycin D (MP Biomedicals, Solon, OH, USA) (final concentration). One day before experiments, plates were spread with OP50, and were left at RT overnight. Animals were cultivated on the plates for 2 h at 20°C before spaced training, or for 4 h at 20°C before massed training. Under the conditions, ∼50% of protein synthesis of the animal was inhibited by this treatment (Amano and Maruyama, 2011).

### Motility Assay

Motility of wild-type animals treated with inhibitors as described above was examined. Animals were collected in ∼1.0 ml of 0.25% aqueous gelatin solution, and were placed on an agar plate consisting of 1.5% agar, 1.0 mM CaCl_2_, 1.0 mM MgSO_4_, and 5 mM potassium phosphate (pH 6.0). A Kimwipe wick was used to remove as much solution as possible. Once animals started moving in a forward direction, the number of body bends during 10 s was counted for 20 animals. Since *C. elegans* moves forward using a stereotypical sine wave from head to tail, one sine wave was counted as one body bend (Supplementary Table S1).

In the same way, motility of wild-type and mutants (20 animals each) was also analyzed after being conditioned with either 100% EtOH and 160 mM KCl, or 0.01% 1-nonanol diluted with EtOH and 160 mM KCl by massed training or spaced training (Supplementary Table S2).

### Statistical Analysis

Statistical analysis of data was performed using Microsoft^®^ Excel 2011 for Macintosh^®^ with the add-in software Statcel3 (OMS Publ., Saitama, Japan). All data were checked for normality of distribution and homogeneity of variance using χ^2^ goodness of fit test (*p* < 0.05), and were evaluated using Student’s *t-*test for comparisons between pairs of groups, or one-way analysis of variance (ANOVA) for multiple comparisons between groups. If ANOVA results were significant (*p* < 0.05), the Tukey-Kramer *post hoc* test was used. Results are reported as mean ± the standard error of the mean (SEM).

## Results

### Appetitive Conditioning of *C. elegans* with 1-Nonanol and KCl

*Caenorhabditis elegans* innately avoided an olfactory cue, 1-nonanol, and was attracted to KCl on an agar plate assay (Supplementary Figure S1) as previously observed (Ward, 1973; Bargmann et al., 1993). Animals were repelled in a dose-dependent manner by 1-nonanol concentrations ranging from 0.01 to 10% (Supplementary Figure S1C), and were maximally attracted by KCl ranging from 50 to 200 mM (Supplementary Figure S1B). Using 1-nonanol and KCl as a CS and a US, respectively, we developed classical conditioning protocols to study associative learning and memory in *C. elegans*. We chose 0.01% 1-nonanol, diluted with EtOH, as a CS, since 0.01% is the lowest concentration of 1-nonanol to which the animals could respond in a dose-dependent manner (Supplementary Figure S1C). 160 mM KCl was used as a US since osmolality (300 mOSM) of the solution is close to that of culture buffer and media for *C. elegans*. Animals repeatedly treated with both 0.01% 1-nonanol, which was diluted with 100% EtOH, and 160 mM KCl by massed training (without an ITI) were not repelled from 1-nonanol, since their CI values were not significantly higher than the “0” base line. Furthermore, animals repeatedly treated with the chemicals by spaced training (with a 10-min ITI) were attracted to 1-nonanol, since the CI values were significantly higher than the base line (**Figures 1B,C**). Reference animals treated by massed training or spaced training with 0.01% 1-nonanol and 160 mM KCl (unpaired with ISI, 120 s), or with 0.01% 1-nonanol alone, 160 mM KCl alone, 100% EtOH alone, or 100% EtOH and 160 mM KCl avoided 1-nonanol, and their CIs were not statistically different from that of naïve animals in the chemotaxis assay (**Figures 1B,C**). Wild-type animals’ sensitivity to 1-nonanol and 160 mM KCl, and their locomotion were not affected by the chemical treatments during massed or spaced training (**Figures 1B,C** and Supplementary Table S2). These results demonstrated that animals conditioned by the massed training using 1-nonanol as a CS and KCl as a US associated the two signals, and were not repelled by 1-nonanol, and that animals conditioned by the spaced training switched their behavior from repulsion to attraction in response to 1-nonanol stimulation.

### Optimal ISI, ITI and Training Cycle Numbers for Memory Retention

We studied the effect of ISIs on memory retention to optimize the conditioning protocols. “Forward conditioning,” in which presentation of a CS precedes that of a US, often produces optimal conditioning (e.g., Jones, 1962; Schneiderman and Gormezano, 1964; Hawkins et al., 1986), while “backward conditioning” can also successfully induce the same memory to those by “forward conditioning” (Dostalek, 1976; Spetch et al., 1981; Durkovic and Damianopoulos, 1986; Amano and Maruyama, 2011). Alternatively, “backward conditioning” in which an aversive US precedes a CS establishes a conditioned approach to the aversive stimulus as a signal for “relief” (e.g., Tanimoto et al., 2004; Andreatta et al., 2012; for a review, see Gerber et al., 2014). Animals that were backwardly conditioned with 160 mM KCl first and then with 0.01% 1-nonanol severely twitched, and could not crawl on the agar surface. Therefore, we tested the effect of ISIs on learning and memory only by “forward conditioning.” Consecutive conditioning with 1-nonanol and KCl without an intervening delay most efficiently induced memory, and the efficiency decreased exponentially as the ISIs increased (**Figure 2A**). Almost no memory was formed with ISIs ≥60 s, consistent with our previous results in aversive olfactory conditioning (Amano and Maruyama, 2011).

We also analyzed effects of ITIs on memory retention. Animals were given eight cycles of conditioning, of which ITI lengths ranged from 0 min through 30 min. Memory retention was analyzed by measuring LI values immediately and 6 h after training. When assayed immediately after training, there were no statistically significant differences among LIs, except for those of animals trained with a 30-min ITI (**Figure 2B**). When assayed 6 h after training, in contrast, LIs of trained animals were elevated as the ITI lengths were increased up to 10 min, and then the LIs gradually decreased when it was longer than 10 min. These results demonstrate that the 10-min ITI is most efficient for animals to retain the memory for 6 h after training, and are consistent with those of aversive olfactory conditioning (Amano and Maruyama, 2011).

These results demonstrated that consecutive conditioning (ISI, 0 s) with 0.01% 1-nonanol and 160 mM KCl efficiently induced associative memory. Associative memory induced by spaced training with a 10-min ITI was most efficiently retained at 6 h after the training under the conditions tested. Therefore, using consecutive spaced training (ISI, 0 s) with a 10-min ITI, we examined the effect of conditioning cycle numbers with 0.01% 1-nonanol and 160 mM KCl on memory formation. Spaced training with >7 cycles induced statistically significant (*p* < 0.01) enhancement of memory formation in comparison to single conditioning, demonstrating that repeated conditioning is required for efficient induction of associative memory (**Figure 2C**).

### Memory Retention and Extinction

**FIGURE 3:**
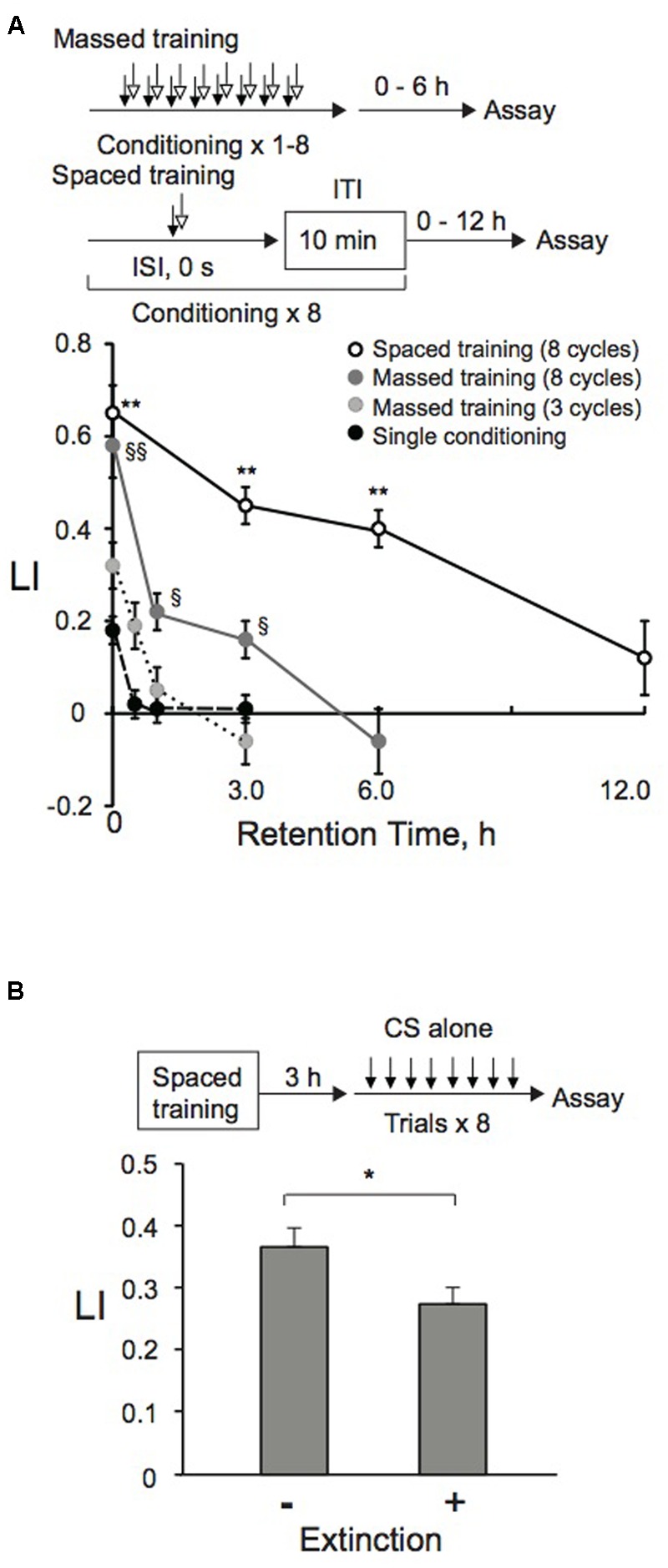
**Memory formation and retention after massed, spaced, or extinction training. (A)** Memory retention curve. Animals were conditioned with 0.01% 1-nonanol and 160 mM KCl indicated numbers of cycles by massed or spaced training. Their LIs were measured at indicated time intervals after conditioning. Asterisks indicate statistically significant differences (^∗∗^*p* < 0.01) determined by one-way ANOVA, followed by the Tukey-Kramer test for further comparison of LIs of animals conditioned by spaced training with those of animals conditioned by single conditioning or massed training. However, LIs of animals conditioned by eight-cycle spaced training or massed training were statistically indistinguishable from each other, when analyzed by using a two-sided Student’s *t*-test. LIs of animals conditioned by the eight-cycle massed training showed statistically significant differences (^§^*p* < 0.05, ^§§^*p* < 0.01) determined by one-way ANOVA, followed by the Tukey-Kramer test for further comparison of LIs of animals conditioned by single conditioning or three-cycle massed training. LI values were calculated from the equation shown in **Figure 2A** using CI_reference_ values of animals treated with 100% EtOH and 160 mM KCl. Data are means ± SEM (*n* = 6–9 assays). **(B)** Extinction learning after spaced training. Animals were conditioned with 0.01% 1-nonanol and 160 mM KCl by eight-cycle spaced training with a 10-min ITI. They were then transferred to an NGM plate with a bacterial lawn. After 3 h incubation at 20°C, animals were treated eight times (without ITI) only with 0.01% 1-nonanol diluted with EtOH or 100% EtOH. Immediately after the treatment, chemotaxis assays were performed. LIs of animals with and without extinction trials were statistically different (^∗^*p* < 0.05) when analyzed using a two-sided Student’s *t-*test. LI values were calculated from the equation shown in **Figure 2A** using CI_reference_ values of animals treated with 100% EtOH and 160 mM KCl. Data are displayed as means ± SEM (*n* = 9 assays).

With optimized ISI and ITI lengths, as well as with optimal conditioning trial numbers, we also analyzed retention time of memory induced by massed or spaced training. After completion of training, animals were transferred to NGM plates with bacteria, where they were allowed to move and eat at 20°C for various time intervals until post-training chemotaxis assay. LI values induced by eight-cycle massed and spaced training were statistically indistinguishable when assayed immediately after training (**Figure 3A**). However, memory induced by massed training was no longer observable at 6 h. LI values after single conditioning or three-cycle massed training were statistically smaller than those induced by eight-cycle massed training when assayed immediately after conditioning, and the memory became unobservable more rapidly than those after eight-cycle massed training. Memory induced by eight-cycle spaced training was observable beyond 6 h after conditioning as consolidated LTM shown below. After consolidation of associated memory between CS and US, further presentations of the CS alone (reactivation) can destabilize it. Reactivation can lead to extinction, a decrease of the response resulting from new associative memory between CS and no US (Myers and Davis, 2002; Suzuki et al., 2004). When animals conditioned by spaced training using 1-nonanol and KCl were exposed to 1-nonanol alone, their LI values were lower than those of animals stimulated with EtOH as a negative control (**Figure 3B**), suggesting that extinction learning occurs in *C. elegans*. This decrease is not due to habituation or adaptation, since chemotactic activity of animals exposed repeatedly to the CS alone or to 100% EtOH is similar to that of naïve animals (**Figure 1B**).

### Sensitivity of Memory to Retrograde Amnesia

**FIGURE 4:**
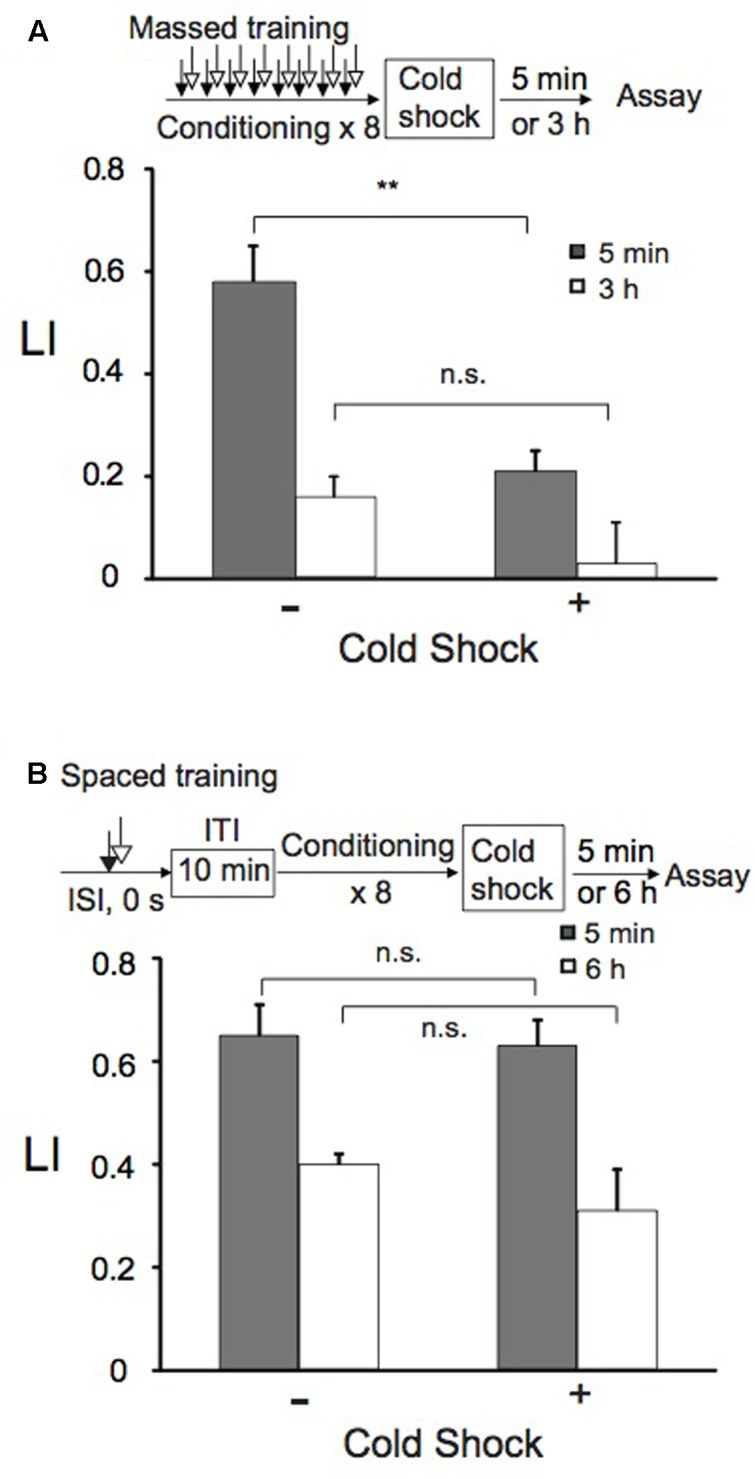
**Short-term memory (STM), but not LTM, is disrupted by cold shock.** Animals were conditioned with 0.01% 1-nonanol and 160 mM KCl by eight-cycle massed training **(A)** or eight-cycle spaced training with a 10-min ITI **(B)**. Immediately after conditioning, animals were immersed in ice-cold ddH_2_O for 5 s as cold shock. Chemotaxis assays were performed 5 min (gray bars) and 3 or 6 h (open bars) after the recovery from cold shock. Note that only LI values of animals with and without cold shock after massed training were statistically different (^∗∗^*p* < 0.01) and that all the other LIs, which include LIs 3 h after massed training, with cold shock were statistically indistinguishable from those without cold shock, when analyzed using a two-sided Student’s *t-*test. LI values were calculated from the equation shown in **Figure 2A** using CI_reference_ values of animals treated with 100% EtOH and 160 mM KCl. Data are displayed as means ± SEM (*n* = 6–9 assays). n.s., not significant.

Short-term memory is vulnerable to disruption by factors such as anesthesia and cold shock (Tully et al., 1994). Therefore, we examined whether memory induced by massed training or spaced training is sensitive to cold shock. Immediately after massed training or spaced training, animals were treated with cold shock by immersing them in ice-cold ddH_2_O for 5 s. After recovering at RT for 5 min or 3 h (massed training), or for 5 min or 6 h (spaced training) on NGM plates with bacteria, animals were assayed for chemotaxis to 1-nonanol. Cold shock did not affect memory retention induced by spaced training, while LI after massed training was markedly decreased (**Figure 4**). Of the memory formed by massed training, approximately 2/3 was cold shock-sensitive memory and the rest was cold shock-resistant memory, when assayed 5 min after cold shock. Cold shock did not significantly affect memory retained at 3 h after massed training. This may be partly because the memory decayed quickly even without cold shock. Since memory after spaced training was resistant to cold shock, it might be consolidated during repetitive conditioning with a 10-min ITI.

### Effect of Translation and Transcription Inhibitors on Memory Formation

**FIGURE 5:**
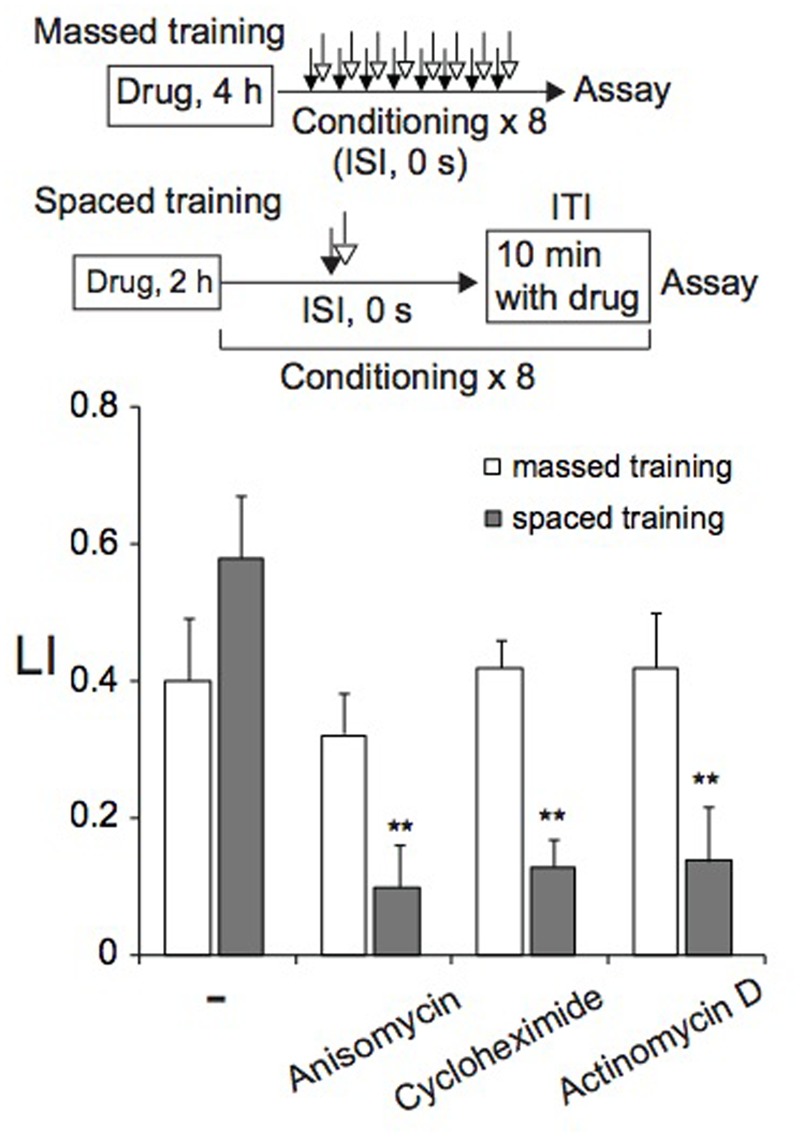
***De novo* translation and transcription are required for LTM, but not for STM.** Animals were cultured on agar plates containing anisomycin, cycloheximide, or actinomycin D for 4 h (massed training) or 2 h (spaced training) at RT, and were then conditioned with 0.01% 1-nonanol and 160 mM KCl by eight-cycle massed training (open bars) or eight-cycle spaced training with a 10-min ITI (gray bars). Chemotaxis assays were performed immediately after training. LI values were calculated from the equation shown in **Figure 2A** using CI_reference_ values of unpaired animals treated with 0.01% 1-nonanol and 160 mM KCl (ISI, 120 s). Asterisks (^∗∗^) indicate statistically significant differences (*p* < 0.01) determined by one-way ANOVA, followed by the Tukey-Kramer test for further comparison with LIs of untreated animals. Bars are means ± SEM (*n* = 6–9 assays).

As described above, memory induced by spaced training was resistant to cold shock, suggesting that it might be LTM, formation of which can be inhibited by treatments of animals with transcription and translation inhibitors (Flexner et al., 1962; Agranoff and Klinger, 1964; Barondes and Jarvik, 1964; Flood et al., 1973; Mizumori et al., 1987; Bourtchuladze et al., 1994; Tully et al., 1994; Epstein et al., 2003; Jarome and Helmstetter, 2014). Therefore, we examined the effect of a translation inhibitor, anisomycin or cycloheximide, and a transcription inhibitor, actinomycin D, on memory formation by massed or spaced training. Before spaced training, animals were cultivated for 2 h on NGM plates spread with bacteria in the presence of 0.3 μg/ml anisomycin, 0.3 μg/ml cycloheximide, or 0.1 μg/ml actinomycin D (final concentration), and then during ITI, animals were placed on NGM plates spread with bacteria that contained the inhibitor. Therefore, animals were cultivated on NGM plates containing the inhibitor for ∼3.2 h in total. Under these conditions, approximately 50% of protein synthesis in *C. elegans* was inhibited (Amano and Maruyama, 2011). Inhibitor-treated animals conditioned by spaced training formed associative memory between 1-nonanol and KCl less effectively than untreated animals, indicating that both translation and transcription are required for memory formation (**Figure 5**). In contrast, memory formation after massed training required neither translation nor transcription, since memory was normally induced in animals cultivated on NGM plates in the presence of the inhibitor for 4 h before training started. Inhibitor treatment of animals under the conditions used affected neither motility (Supplementary Table S1), nor chemotaxis to 1-nonanol or to 160 mM KCl (Supplementary Figure S2). In the experiments of **Figure 5**, LI values were calculated by subtracting of CI of unpaired (ISI, 120 s) animals stimulated with 1-nonanol and KCl from that of paired (ISI, 0 s) animals. Together with the results of cold-shock sensitivity described above, these results indicate that memories induced by massed and spaced training are STM and LTM, respectively.

### *C. elegans* Mutants Defective in STM and/or LTM

**FIGURE 6:**
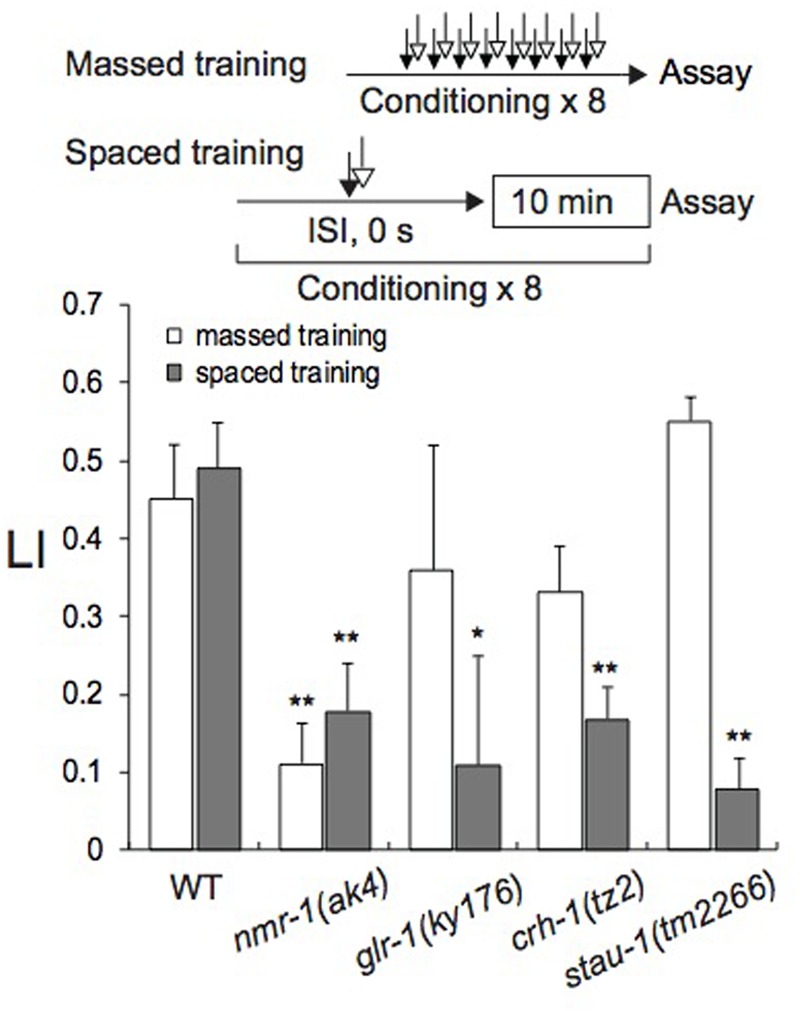
***Caenorhabditis elegans* mutants defective in learning and memory.** Wild-type N2 and mutants were conditioned with 0.1% 1-nonanol and 160 mM KCl using eight-cycle massed training (open bars) or eight-cycle spaced training with a 10-min ITI (gray bars). Chemotaxis assays were performed immediately after conditioning. LI values were calculated from the equation shown in **Figure 2A** using CI_reference_ values of unpaired animals treated with 0.1% 1-nonanol and 160 mM KCl (ISI, 120 s). Asterisks indicate statistically significant differences (^∗^*p* < 0.05, ^∗∗^*p* < 0.01) of mutant LIs in comparison with those of the wild-type, using two-sided Student’s *t-*test. Data are displayed as means ± SEM (*n* = 6–9 assays).

The *C. elegans* genome encodes “learning and memory genes,” including *crh-1* for the ubiquitous transcription factor CREB (cAMP-responsive element-binding protein), *glr-1* for α-amino-3-hydroxy-5-methyl-4-isoxazolapropionic acid (AMPA)-type, and *nmr-1* for *N*-methyl-D-aspartate (NMDA)-type glutamate receptor subunits. Previous studies in *Aplysia, C. elegans, Drosophila*, and mice (e.g., Dash et al., 1990; Dubnau et al., 2003; Hobert, 2003; Xia et al., 2005; Kano et al., 2008; Kauffman et al., 2010; Amano and Maruyama, 2011; Miyashita et al., 2012; Sasakura and Mori, 2013; Shipton and Paulsen, 2014) have shown that these genes play vital roles in classical conditioning. *stau-1*, which encodes the double-stranded RNA-binding protein Staufen isoform (LeGendre et al., 2013), has been shown to play crucial roles in classical conditioning in *C. elegans* and *Drosophila* (Dubnau et al., 2003; Amano and Maruyama, 2011). Therefore, we also examined whether the gene is involved in memory formation after massed or spaced training. In chemotaxis assays, all mutants as well as wild-type N2 avoided 1-nonanol, but sensitivities of *nmr-1(ak4)* and *crh-1(tz2)* to 0.01% 1-nonanol were significantly lower than that of the wild-type (Supplementary Figure S3A). Therefore, 0.1% 1-nonanol, which produced similar CI values for all strains analyzed, was used for classical conditioning and the chemotaxis assay. All the mutants were attracted to 160 mM KCl at statistically indistinguishable efficiencies from that of wild-type (Supplementary Figure S3B). Motility of mutant strains before and after training was statistically indistinguishable from that of wild-type (Supplementary Table S2). In the experiments of **Figure 6**, LI values were calculated by subtracting of CI of unpaired (ISI, 120 s) animals stimulated with 1-nonanol and KCl from that of paired (ISI, 0 s) animals.

In comparison to wild-type, *nmr-1(ak4)* formed both STM and LTM less effectively after massed and spaced training with 0.1% 1-nonanol and 160 mM KCl (**Figure 6**). By contrast, *crh-1(tz2), glr-1(ky176)*, and *stau-1(tm2266)* were successfully conditioned by massed training, but less effectively formed LTM than the wild-type after eight-cycle spaced training. These results demonstrate that only *nmr-1* among the investigated genes is required for STM, and all genes examined are essential for LTM. No effect of *glr-1(ky176) and stau-1(tm2266)* in the formation of STM is discussed below.

## Discussion

In the present study we developed a classical conditioning paradigm to study associative learning and memory in *C. elegans*. This appetitive olfactory conditioning with 1-nonanol and KCl, used as a CS and a US, respectively, shares many of the defining features of associative learning in vertebrate and invertebrate species, as exemplified by classical conditioning. These include contiguity learning, and both short-term and long-term retention of memories. Furthermore, it is also possible to extinguish learned behavior to some extent by extinction learning, in which presentation of a reinforcing stimulus is withheld.

Short-term memory and LTM have successfully been induced with massed training and spaced training, respectively. The only difference between the two training protocols is an ITI, which is also called “a resting interval,” between trials in spaced training. LTM formation is dependent on mRNA and protein synthesis, while STM is not. These are the major features of LTM and STM (e.g., Flexner et al., 1962; Agranoff and Klinger, 1964; Barondes and Jarvik, 1964; Stanton and Sarvey, 1984; Bourtchuladze et al., 1994; Nguyen et al., 1994; Tully et al., 1994; Crow et al., 1997; Epstein et al., 2003; Fulton et al., 2005; Kauffman et al., 2010; Amano and Maruyama, 2011). When *C. elegans* was conditioned with butanone as a CS and bacterial food as a US, STM and LTM were formed (Kauffman et al., 2010). A single treatment of the animal, which was starved for an hour, with 10% butanone and food for 30 min successfully induced STM, which was no longer observable after 2 h. The single trial also induced LI values similar to those measured immediately after seven-cycle spaced training, although six or seven training sessions were required to retain the memory for 16 or 24 h, respectively. In contrast, in the present study using 1-nonanol and KCl, memory induced by eight-cycle spaced training was retained less than 12 h, and a single trial induced only ∼30% of maximum memory induced after eight-cycle massed training (**Figure 3A**). These differences may be derived from either a distinct US (food or KCl) or the duration of training per cycle (30 min vs. 10 s).

In general, when presentation of a CS precedes that of a US by a brief interval, optimal conditioning is observed (“forward conditioning”) (Jones, 1962; Schneiderman and Gormezano, 1964; Hawkins et al., 1986). There are also examples of classical conditioning that simultaneous pairing is as effective, or more effective than forward pairing (e.g., Mahoney and Ayres, 1976; Rescorla, 1980; Tully and Quinn, 1985; Barnet et al., 1991, 1993; Lin and Glanzman, 1997; Lent and Kwon, 2004; Amano and Maruyama, 2011). Consistent with these cases, the present results demonstrate that the most efficacious procedure for appetitive olfactory conditioning is to have consecutive presentation of CS and US without an intervening delay (**Figure 2A**).

The memory augmentation induced by spaced training is called the spacing effect, which is a common phenomenon in animals, including humans (Carew et al., 1972; Gerber et al., 1998; Rose et al., 2002; Cepeda et al., 2006). The optimal ITI was determined to be ∼10 min for 6-h retention of LTM (**Figure 2B**). This is similar to optimal ITIs of other organisms, including *Drosophila*, honeybees and crickets (Beck et al., 2000; Menzel et al., 2001; Matsumoto and Mizunami, 2002; Giurfa et al., 2009; Rohwedder et al., 2015). The spacing effect has long been known at the behavioral level, but the underlying cellular and molecular mechanisms are not well-understood. Mitogen-activated protein kinase (MAPK) activity has been implicated in memory formation in vertebrates and invertebrates (Kandel, 2001; Kelleher et al., 2004; Mayford, 2007; Cammarota et al., 2008), and recent studies suggest that activation of MAPK during ITIs is necessary for LTM (Ye et al., 2008; Pagani et al., 2009; Li et al., 2016).

We have also analyzed effects of mutations on formation of STM and LTM (**Figure 6**). *nmr-1(ak4)* was defective in formation of both STM and LTM. Mutations in *crh-1(tz2), glr-1(ky176)*, and *stau-1(tm2266)* affected only LTM. In *C. elegans, nmr-1* is expressed only in six pairs of neurons (AVA, AVD, ADE, RIM, AVG, and PVC) (Brockie et al., 2001a,b). In these neurons, the NMDA receptor may act as a molecular coincidence detector for 1-nonanol and KCl signals for synaptic plasticity, where synaptic strengthening required for both STM and LTM can result from coincidental firing of pre- and post-synaptic neurons (Gustafsson and Wingstrom, 1988; Kauer et al., 1988; Bliss and Collingridge, 1993; Bailey et al., 2000; Miyashita et al., 2012). Influx of Ca^2+^ through glutamate receptors into post-synaptic cells can induce activation of protein kinases such as MAPK and CaMKII (Chen et al., 1998; Bailey et al., 2000; Wang et al., 2007), which phosphorylate the transcription-factor CREB. CREB, encoded by *crh-1* (Kimura et al., 2002; Suo et al., 2009; Nishida et al., 2011; Timbers and Rankin, 2011), is a member of the basic region/leucine zipper (bZip) family of transcription factors. It is regulated by increases in intracellular levels of cAMP and Ca^2+^ (Carlezon et al., 2005), and activates a cascade of genes that leads to LTM (Dash et al., 1990; Yin et al., 1994; Barco and Marie, 2011; Kida and Serita, 2014; Lakhina et al., 2015). The *glr-1* gene encodes one subtype of ionotropic glutamate receptor channels, and is critical for LTM formation. Its expression and localization altered by conditioning are necessary for formation of long-term habituation in *C. elegans* (Rose et al., 2005).

The phenotypic difference between *nmr-1* and *glr-1* may be due to the number of homologs encoded by the genome. Six of the subunits, including four non-NMDA (*glr-1, glr-2, glr-4*, and *glr-5*) and two NMDA (*nmr-1* and *nmr-2*), are expressed in many of the command interneurons, which control forward and backward locomotion (Brockie and Maricq, 2006). During massed training, redundant GLR activities might compensate the missing or reduced activity of GLR-1, while full activity of all the GLR subunits may be required for LTM. During spaced training, activity of redundant homologs may not be strong enough to compensate for the missing or reduced activity of one of the homologs for learning and memory. Similarly, *stau-1(tm2266)* is a partial loss-of-function mutant (LeGendre et al., 2013), and the reduced activity may be sufficient for STM but not for LTM.

## Conclusion

The appetitive olfactory conditioning with 1-nonanol and KCl shares many features of associative learning observed in other invertebrate and vertebrate. These include contiguity learning, and both short-term and long-term retention of memories. Furthermore, the learned behavior can be extinguished to some extent by extinction learning, in which presentation of reinforcing stimuli is withheld. The formation of LTM, but not STM, was dependent on mRNA and protein synthesis, and required activity of genes shared by other model organisms, including *Aplysia, Drosophila*, and mice.

Our previous (Amano and Maruyama, 2011) and present studies have now established aversive and appetitive conditioning paradigms with combinations of two defined chemical cues, which may allow us to elucidate neuronal circuit plasticity for learning and memory in *C. elegans*. Analysis of the two paradigms may give insights into their similarities and differences at neuronal circuit and molecular levels.

## Author Contributions

SN and IM conceived and designed the experiments. SN performed the experiments. SN and IM analyzed the data, and wrote and edited the manuscript.

## Conflict of Interest Statement

The authors declare that the research was conducted in the absence of any commercial or financial relationships that could be construed as a potential conflict of interest.
