# Supplementary material for: Appetitive Olfactory Learning and Long-Term Associative Memory in Caenorhabditis elegans
Source: Front Behav Neurosci. 2017 May 1;11:80. doi: 10.3389/fnbeh.2017.00080 (PMC5410607; doi:10.3389/fnbeh.2017.00080)
Supplement: Supplementary file 1 [file Table_1.PDF]

Supplementary Table S1

Motor activity of animals after inhibitor treatment

Inhibitor	Number of body bends* /10 s (mean ± SEM)**
-	10.2 ± 0.3
Anisomycin	9.6 ± 0.3
Cycloheximide	9.8 ± 0.2
Actinomycin D	9.8 ± 0.2

*20 animals each treated with the inhibitor were analyzed.

**No statistically significant differences among the data when analyzed by using one-way ANOVA.
