# Supplementary material for: Appetitive Olfactory Learning and Long-Term Associative Memory in Caenorhabditis elegans
Source: Front Behav Neurosci. 2017 May 1;11:80. doi: 10.3389/fnbeh.2017.00080 (PMC5410607; doi:10.3389/fnbeh.2017.00080)
Supplement: Supplementary file 2 [file Table_2.PDF]

Supplementary Table S2

Motor activity of animals after massed or spaced training

Massed training	Body bends*/10 s (mean ± SEM)**		
	naive	1-nonanol & KCl	EtOH & KCl
N2	10.2 ± 0.3	10.4 ± 0.2	10.7 ± 0.3
glr-1(ky176)	9.0 ± 0.4	9.1 ± 0.4	9.6 ± 0.5
nmr-1(ak4)	11.0 ± 0.2	11.0 ± 0.2	11.0 ± 0.3
crh-1(tz2)	10.1 ± 0.5	9.7± 0.3	9.2 ± 0.5
stau-1(tm2266)	11.2 ± 0.5	10.8 ± 0.4	11.5 ± 0.3

Spaced training	Body bends*/10 s (mean ± SEM)**		
	naive	1-nonanol & KCl	EtOH & KCl
N2	10.2 ± 0.3	10.4 ± 0.2	10.2 ± 0.3
glr-1(ky176)	9.0 ± 0.4	9.0 ± 0.3	9.3 ± 0.4
nmr-1(ak4)	11.0 ± 0.2	10.7 ± 0.3	10.5 ± 0.3
crh-1(tz2)	10.1 ± 0.5	10.2 ± 0.4	10.4 ± 0.4
stau-1(tm2266)	11.3 ± 0.3	10.8 ± 0.4	11.0 ± 0.4

Note that 0.1% 1-nonanol was used for these experiments.
*20 animals of each strain were analyzed.
**No statistically significant differences among the data in the categories when analyzed by using one-way ANOVA.
