# Supplementary material for: Appetitive Olfactory Learning and Long-Term Associative Memory in Caenorhabditis elegans
Source: Front Behav Neurosci. 2017 May 1;11:80. doi: 10.3389/fnbeh.2017.00080 (PMC5410607; doi:10.3389/fnbeh.2017.00080)
Supplement: Supplementary file 3 [file Presentation_1.PDF]

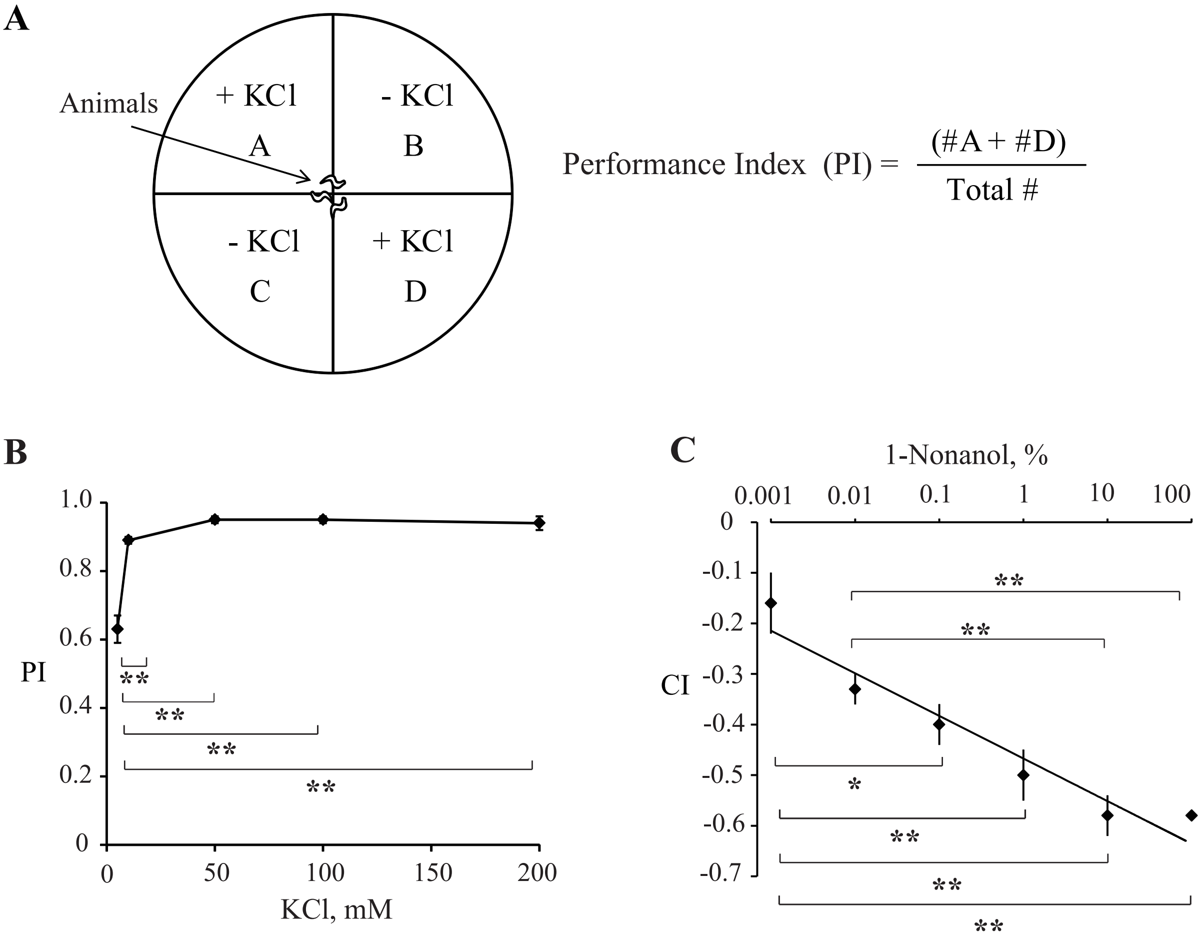

Supplementary Figure S1. Sensitivity of wild-type animals to KCl and 1-nonanol.

(**A**) Schematic representation of a 10-cm Petri dish, and an equation used for resource localization assay. Ingredients of agar plates are described in Materials and Methods of main text.
(**B**) Sensitivity of wild-type animals to various concentrations of KCl. The sensitivity was analyzed by using a Petri dish divided into four quadrants shown in (A), as described in Materials and Methods of main text. Approximately 100 animals were placed at the intersection of the four quadrants. After 30 min, animals were killed by placing of chloroform on the lid and the number of animals in each four quadrants was counted. PI values were calculated using the equation shown in (A). (**C**) Sensitivity of wild-type animals to various concentrations of 1-nonanol. Square-plate chemotaxis assay was used as described in Figure 1, and Materials and Methods of main text. Asterisks indicate statistically significant (*$p < 0.05$, **$p < 0.01$) differences determined by one-way ANOVA, followed by the Tukey-Kramer test for further pair-wise comparisons of all the data points. Data are displayed as mean ± SEM (n = 6-9 assays).
