# Supplementary material for: Appetitive Olfactory Learning and Long-Term Associative Memory in Caenorhabditis elegans
Source: Front Behav Neurosci. 2017 May 1;11:80. doi: 10.3389/fnbeh.2017.00080 (PMC5410607; doi:10.3389/fnbeh.2017.00080)
Supplement: Supplementary file 4 [file Presentation_2.PDF]

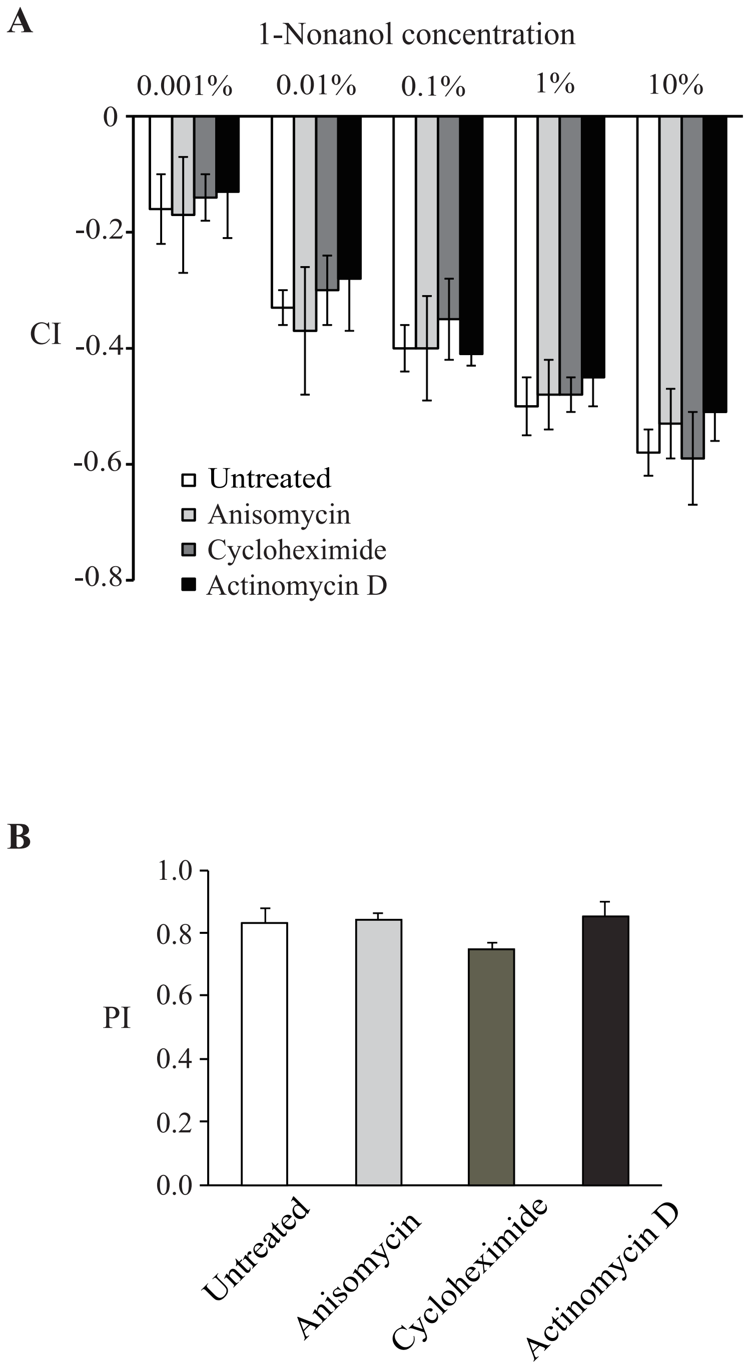

Supplementary Figure S2. Effects of inhibitors on animal's sensitivity to 1-nonanol and KCl. (**A**) Sensitivity of wild-type animals treated with translation or transcription inhibitors to various concentrations of 1-nonanol. Animals were treated with 0.3 μg/ml of anisomycin, 0.3 μg/ml cycloheximide, or 0.1 μg/ml actinomycin D, and their sensitivity to indicated concentrations of 1-nonanol was analyzed by square-plate chemotaxis assay as described in Materials and Methods of main text. No statistical differences among data points in each group when analyzed by one-way ANOVA. (**B**) Sensitivity of wild-type animals treated with the inhibitor to 160 mM KCl. Animals were treated with the inhibitor as described in Materials and Methods of the main test, and their performance index (PI) was measured by using resource localization assay as described in Materials and Methods and Supplementary Figure S1. No statistical differences among data points when analyzed by one-way ANOVA. Data are displayed as mean ± SEM (*n* = 3-9 assays).
