# Supplementary material for: Appetitive Olfactory Learning and Long-Term Associative Memory in Caenorhabditis elegans
Source: Front Behav Neurosci. 2017 May 1;11:80. doi: 10.3389/fnbeh.2017.00080 (PMC5410607; doi:10.3389/fnbeh.2017.00080)
Supplement: Supplementary file 5 [file Presentation_3.PDF]

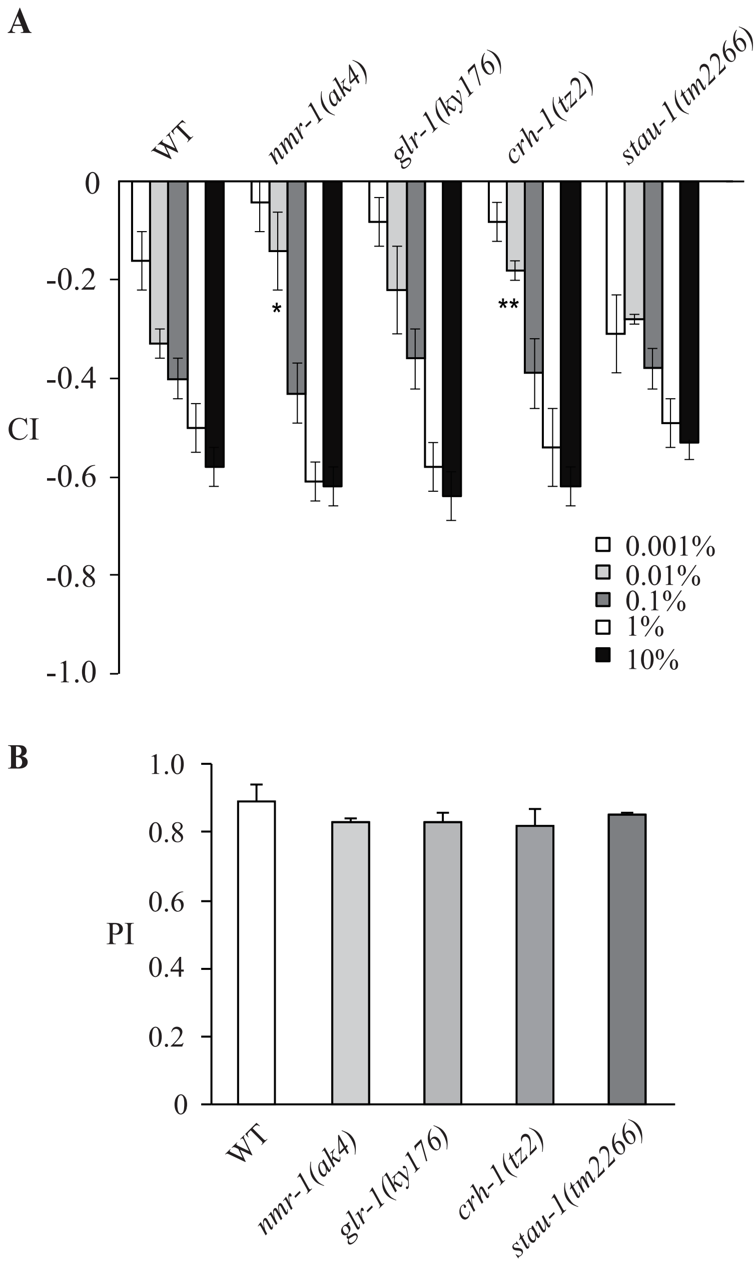

Supplementary Figure S3. Sensitivity of mutants to 1-nonanol and KCl. (**A**) Sensitivity of mutants to various concentrations of 1-nonanol. CI values of mutants were measured by square-plate chemotaxis assay as described in Materials and Methods of main text. Asterisks indicate statistically significant (*p < 0.05, **p < 0.01) differences determined by two-sided Student's *t*-test in comparison with CI values of wild-type (WT) animals. (**B**) Sensitivity of mutants to 160 mM KCl by using resource localization assay as described in Materials and Methods of main text and Supplementary Figure S1A. No statistical differences are determined among data points by using one-way ANOVA. Data are displayed as means ± SEM (n = 9 assays).
